# 

**Published:** 1893-03

**Authors:** 


### LITERARY.

THE MAYBRICK CASE.—It is highly creditable that the women of America have taken so deep an interest in the criminal prosecution of one of their sex for a cruel and infamous crime in an English Court of Justice, and her sentence after conviction to the death penalty, subsequently commuted to imprisonment for life.

The pamphlet before us of 150 pp., by Dr. Helen Densmore, is a thorough review of all the circumstances and proceedings in this nineteenth century travesty of justice before an imbecile judge, who in the face of inadequate proof, lent the powers of his high office to convict an American woman of a crime of which, by all legal rule, she should have been found guiltless : a miscarriage of justice which it would be difficult to parallel in the published records of the two countries wherein trial by jury prevails.

The lukewarmness of the Home Office in which the pardoning power is lodged, is a mystery which calls for a better explanation than any thus far made. Neither human life nor human liberty should be held in such light account where English ideas are cherished and English laws prevail.

The philanthropic author of this exhaustive review in behalf of a woman falsely accused and unfairly tried and convicted, devotes the entire proceeds of its sale to the Maybrick fund in aid of justice. It is a case in which every woman of this country should have an interest, and if the case is unfamiliar to one, this pamphlet sold at 25 cents will explain everything.

Address Stillman & Co., 1398 Broadway, N. Y.

HOW NATURE CURES.—Comprising a New System of Hygiene, also The Natural Food of Man. This is a 4to of 413 pp., including a complete index, by Emmet Densmore, M.D. Stillman & Co., 1398 Broadway, Publishers.

This interesting volume is in three parts: 1st, "How to Doctor ;" 2d, "How to get well and keep well;" 3d, "The Natural Food of Man."

The author in his preface quotes as a comprehensive text to all that follows, the following epigrammatic statement from an essay by Mrs. Helen Densmore, to whom his work is dedicated.

"Health is man's birthright. It is as natural to be well as to be born. All pathological conditions, all diseases, and all tendencies to disease are the result of the transgression of physiological and hygienic law, this is the science of health in a nutshell."

It is the amplification of the foregoing text to which the volume in question is devoted. All starch bearing foods are disparaged. This includes potatoes, and the cereals, which constitute the main dependence of man for subsistence. Fruit and nuts are recommended as nearest to nature and nature's demands, but the difficulty of the food supply, if confined to these ingredients, is apparent to the author. It would be comparatively easy to adopt his theories in countries where fruits of various kinds grow profusely, spontaneously and require but little culture, but in a climate like ours it would hardly be practicable.

We anticipated in this work a wholesale condemnation of a flesh diet, but the author, under certain conditions approves of it, particularly where corpulency is sought to be reduced or cured as a disease.

The author has no use for drugs, or the M.D's. who administer them. His rules of health are correct living, open air exercise, temperance, rest and sleep, and for all these he gives rules which recommend themselves to the intelligent reader.

The book ought to have a large sale.

Our thanks are due for the following :

STATE COMMISSION OF LUNACY.—Third Annual Report, 1891.

The institution is under the Medical Superintendence of Dr. Selden H. Talcott. This institution has 231 acres of land, with a provision for 675 patients. It is one of the best and best conducted in the State.

TUMOR OF THE LIVER—REMOVAL ATTEMPTED.—By John B. Roberts, M.D., Philadelphia. Also INTRA-CRANIAL NEUREOTOMY of 2d and 3d divisions of Fifth nerve. By same.

ARTERIAL SALINE INFUSION, with Report of Special Cases. By Robert H. M. Dawbarr, M.D.

VICK'S FLORAL GUIDE for 1893, is not a whit behind its predecessors. See advertisement, and write for it, with stamps inclosed.

COSMETIC SURGERY OF THE NOSE, by Prof. John B. Roberts, M.D.

